# The Clinicopathological Correlation of KRAS Mutation and PTEN Expression Status in Primary and Metastatic Colorectal Carcinoma

**DOI:** 10.7759/cureus.53884

**Published:** 2024-02-08

**Authors:** Redir T Hassan, Bashar AL Hassawi, Maysoon Alkazzaz

**Affiliations:** 1 Department of Anatomy, University of Duhok, Duhok, IRQ; 2 Department of Biology, University of Duhok, Duhok, IRQ; 3 Department of Anatomy, University of Mosul, Mosul, IRQ

**Keywords:** p-akt and p65, p-erk, pten, kras, colorectal cancer

## Abstract

Background: Colorectal cancer (CRC) research has identified a consistent loss of PTEN expression in both primary tumors and metastasis, highlighting its potential role in this disease. However, the impact of PTEN on downstream proteins of KRAS mutation, namely p-AKT, p-ERK, and p65 (NFkB), remains unknown. This study aims to explore the inhibitory effect of PTEN on KRAS downstream proteins and its correlation with pathological features in CRC patients.

Methods: From January 1, 2015, to December 31, 2021, 86 CRC cases were collected from governmental and private laboratories in the Duhok province. Formalin-fixed, paraffin-embedded tissue blocks were obtained, and the study involved histopathological analysis, immunohistochemistry of PTEN, AKT, ERK, and P65 markers, and molecular analysis of the KRAS gene.

Results: Among the 86 cases, there were 46 males (53.5%) and 40 females (46.5%), with an equal distribution between right colon and left colon/rectum. Tumors larger than 5cm were observed in 47 cases, predominantly displaying a polypoid or ulcerated growth pattern. Most cases were moderately differentiated adenocarcinomas, with stages II and III being the most prevalent 31 cases (36%) and 34 cases (39.5%) respectively. Significant associations were found between PTEN, ERK expressions, and tumor location in the right colon (P=0.031 and P=0.009 respectively). Tumor size correlated with P65 expression (P=0.042). KRAS mutation showed a positive relationship with the type of tumor growth (P=0.035). Tumor grade increased with KRAS mutations (P=0.043). PTEN expression correlated significantly with ERK and AKT markers (P=0.018 and 0.035 respectively). P65 exhibited an association with KRAS mutation (P=0.034).

Conclusion: The study revealed PTEN expression in association with the inhibition of AKT and ERK, and the absence of KRAS gene mutation. Conversely, PTEN is not expressed with the positively reactive P65 and the presence of KRAS mutation. This study contributes valuable insights into the complex interplay between PTEN expression, KRAS mutation, and downstream signaling pathways in CRC. It suggests potential avenues for further research and therapeutic strategies in the context of CRC treatment.

## Introduction

Colorectal cancer (CRC), with diverse histological and molecular characteristics, is increasingly scrutinized for prognostic predictions and therapeutic insights as our molecular understanding deepens [1]. The order of acquiring crucial mutations during colorectal tumor progression remains unclear, posing questions about the differentiation between short-term and long-term survivors and treatment responsiveness, particularly for antibody-based therapies [2].

The anatomical distribution of CRC varies across primary tumor sites, with the left side (descending colon, sigmoid colon, and rectum) accounting for 69% of cases and the right side (ascending colon, cecum, and transverse colon) for 31% [3]. CRC metastasizes through direct extension, lymphatic spread, portal venous spread to the liver, peritoneal dissemination, and vascular spread to distant organs. The liver is the most frequent metastatic site (50%), followed by intra-abdominal (16%) and intrathoracic (11%) metastatic diseases, with brain metastases occurring in approximately 7% of cases [4].

KRAS, a well-known proto-oncogene, presents gain-of-function mutations in 30% to 40% of colon cancer cases, challenging researchers to develop effective therapies for three decades [5]. Activating p-ERK, p-AKT, and P65 downstream events are associated with RAS, but the most effective clinical target within these pathways remains unclear [6]. The tumor suppressor gene PTEN, crucial for maintaining PI3K homeostasis, inhibits NFκB and AKT in T-cell acute lymphoblastic leukemia [7]. This study was conducted to explore the inhibitory effect of PTEN on KRAS downstream proteins and its correlation with pathological features in CRC patients. And also to investigate the expression of KRAS-downstream proteins (p-ERK, p-AKT, and P65) in colorectal carcinoma harbor mutant and wild-type KRAS.

## Materials and methods

This cross-sectional study was conducted at the Department of Anatomy, Biology, and Histology within the College of Medicine at the University of Duhok. It received approval from the medical ethics committee of the General Directorate of Health in Duhok. A total of 86 CRC cases were collected between January 1, 2015, and December 31, 2021. The study encompassed three key analytical components: histopathology (hematoxylin and eosin staining), immunohistochemistry (IHC), and molecular analysis.

Data collection involved retrieving patient information and clinical data from laboratory archives. All CRC cases with sufficient tumor materials were included in the study. Exclusions were made for cases diagnosed endoscopically as CRC without whole tumor resection samples or those with inadequate information.

The histopathological slides were re-evaluated by two pathologists. All cases were examined to determine the histological grade (well-differentiated, moderately differentiated, poor, or undifferentiated). Staging followed the TNM staging system, recommended by the American Joint Committee on Cancer as the standard for determining the prognosis of patients with this cancer.

Immunohistochemistry

The IHC was performed in Vajeen private hospital Lab in the Duhok City Kurdistan region of Iraq. Formalin-fixed, paraffin-embedded (FFPE) histological sections (4 mm in thickness) were immunostained for (PTEN, AKT1, ERK, and NFkB P65). The analysis was performed with anti-PTEN (clone, 6H2.1 Monoclonal Mouse Anti-Human PTEN, dilution 1/100; Dako USA), anti-AKT1 (clone sc-5298, Monoclonal Mouse Antibody, dilution 1/100; Santa Cruz Biotechnology), anti-ERK1 (clone 12D11 (ab119357), Monoclonal Mouse Antibody, dilution 1/100; abcam UK) and anti- NFkB P65 (clone 572, Monoclonal Mouse Antibody, dilution 1/100; Invitogen by Thermos Fisher Scientific USA).

IHC Scoring

A binary scoring system was employed, where the absence of staining in 90% or more of tumor cells was considered as negative PTEN expression, and the presence of staining in more than 10% of tumor cells was considered as positive PTEN expression [8].

AKT: The categorization of the percentage of cells exhibiting staining was done in the following manner: 0 (no staining), 1 (1-25%), 2 (26-50%), or 3 (51-100%). Staining intensity was classified as 0 (negative), 1 (weak), 2 (intermediate), or 3 (strong). The overall IHC score was computed as the sum of these two parameters, and the samples were grouped into the following categories: (0) negative, (1) weak, (2) moderate, and (3) strong. Tissues with scores ranging from 0 to 3 were considered to have low expression, while those with scores from 4 to 6 were classified as having high expression [9].

ERK: The observers scored the staining intensities of cancer cell nuclei on a scale ranging from absent (0) to strong (3). The counts of stained cancer cell nuclei were classified into categories as follows: 0% (0), below 10% (1), 10-60% (2), or above 60% (3). The resulting intensity scores were multiplied by the corresponding number scores, yielding an overall staining characterization categorized as absent (0), weak (≤1), intermediate (1-3), or strong (3) [10,11].

NF κB/p65 staining on slides was assessed following a recommended protocol: Staining intensity ranged from 0 to 3, with 0 indicating negative, 1 indicating weak, 2 indicating moderate, and 3 indicating strong. The extent of staining, representing the percentage of positive epithelial cells in relation to the entire tumor area, was categorized from 0 to 4: 0 (0%), 1 (≤25%), 2 (26-50%), 3 (51-75%), and 4 (>75%). The final staining score was obtained by adding the staining intensity to the extent of staining. Scores equal to or greater than 3 were considered positive [12,13].

Molecular analysis

Genomic DNA Extraction

Genomic DNA was extracted from FFPE specimens using a DNA extraction kit (Thermofisher, USA), following the manufacturer's instructions with slight adjustments. In brief, the DNA concentration was assessed and quantified using a NanoDrop device (ND-1000, USA). Genomic DNA samples with a (A260-A320)/(A280-A320) ratio greater than 1.7 and a concentration exceeding 40 ng/μL were successfully obtained.

Amplification and Library Preparation

Primer design was conducted for the coding regions of the target genes, specifically the KRAS gene. Four primers were used to designed to amplify the KRAS gene as follows (Table 1):

**Table 1:** KRAS gene primers sequences exons 1, 2, 3 and 4

KRAS gene exons	Forward primer sequences	Reverse primer sequences
Exon 1	TTAACCTTATGTGTGACATGTTCTAA	AGAATGGTCCTGCACCAGTAA
Exon 2	CCAGACTGTGTTTCTCCCTTC	TTTAAACCCACCTATAATGGTGAA
Exon 3	GAAGTAAAAGGTGCACTGTA	AACTATAATTACTCCTTAAT
Exon 4	AGACACAAAACAGGCTCAGGA	TTGAGAGAAAAACTGATATATTAAATGAC

Polymerase chain reactions (PCRs) were performed on isolated genomic DNA samples. Each sample underwent PCR, and the resulting amplicons were amalgamated to form PCR pools, consolidating all individual amplicons within a single tube. During this pooling phase, considerations were given to factors like amplification efficiency and amplicon length. The volume of each PCR was adjusted proportionally to the amplicon's length and inversely proportional to the efficiency of the reaction, both determined through gel electrophoresis.

The PCR pools of each sample were purified with the NucleoFast® 96 PCR kit from MACHEREY-NAGEL GmbH, Düren, Germany. These purified pools underwent quantification and standardization, achieving a concentration of 0.2 ng/μL, a prerequisite for the subsequent sample preparation stage. Sample preparation for next-generation sequencing was executed using the NexteraXT sample preparation kit from Illumina Inc., USA. The Miseq system, also from Illumina Inc., was employed for the next-generation sequencing of the samples. Following sequencing, the obtained data underwent analysis utilizing the IGV 2.3 software developed by the Broad Institute, Cambridge, USA.

Raw sequence reads were aligned to the hg19 reference genome using the bwa mem 0.7.17 alignment tool [14]. Following alignment, additional steps including sorting, duplicate marking, and base recalibration were performed using GATK4 [15]. Variant calling was conducted using two separate algorithms: both GATK UnifiedGenotyper and GATK HaplotypeCaller (Broad Institute, Cambridge, USA) were employed to ensure comprehensive variant detection and complement each other in the process. Low-quality variants identified by both algorithms were filtered out based on parameters such as strand bias, read depth, and call quality using the GATK SelectVariants option [15].

In silico analysis

Various computational tools were employed to forecast the impact of mutations on structural characteristics and protein function. The functionality of variants was assessed using tools like Polymorphism Phenotyping (PolyPhen-2) (Broad Institute, Cambridge, USA) and Sorting Intolerant from Tolerant (SIFT) (J. Craig Venter Institute, Rockville, USA). MutationTaster (University of Tübingen, Tübingen, USA) was utilized to evaluate how mutations affect both protein function and structure. In addition, Align GVGD (University of California, California, USA) was employed to calculate a biochemical distance score.

Statistical analysis

Statistical analyses were meticulously performed using the advanced Statistical Package for the Social Sciences (IBM SPSS Statistics for Windows, IBM Corp., Version 25.0, Armonk, NY), a highly regarded software application renowned for its robust capabilities in the comprehensive examination and interpretation of data sets within the field of social sciences and beyond. To investigate the association between protein expression and clinicopathological factors, as well as between cancer and normal tissues, Fisher's exact or chi-square tests were employed. Statistical significance was ascertained in all analyses based on the convention that the P value, representing the probability of obtaining results as extreme as the observed ones under the assumption of no effect, was considered to be indicative of significance if it fell below the predetermined threshold of 0.05, thereby affirming the validity of the observed findings.

## Results

Clinicopathological features of CRC

Key findings of the clinicopathological features of CRC patients (Table 2) include a higher male prevalence 46 (53.5%) compared to females 40 (46.5%) and a majority of cases (57) in individuals aged ≤50 (66.3%). The most common tumor sites were in the cecum (29 cases) (33.7%), followed by the sigmoid colon (21) (24.4%). Grossly the polypoidal (36) (41.9%) and ulcerated (35) (40.7%) growth types were the most common. Tumor sizes ≤5 cm (54.7%) (47) were slightly more prevalent than those >5 cm (45.3%) (39). Moderate differentiation (67 cases) (77.9%) was most frequent, followed by well-differentiated (7%) (7) and poorly differentiated cases (13) (15.1%).

**Table 2:** Clinicopathological features of colorectal cancer No. = number of cases, % = 100 percentage

Clinicopathological parameters	Variables	No. cases	Percentage (%)
Sex	Male	46	53.5
	Female	40	46.5
Age	≤50	57	66.3
	>50	29	33.7
Location of tumor	Cecum	29	33.7
	Ascending colon	11	12.8
	Transverse colon	3	3.5
	Descending colon	3	3.5
	Sigmoid colon	21	24.4
	Rectum	19	22.1
Type of growth	Polypoid	36	41.9
	Ulcerated	35	40.7
	Circumferential	8	9.3
	Fungating	7	8.1
Size of Tumor	≤5 cm	47	54.7
	>5 cm	39	45.3
Grade	Well-differentiated	6	7
	Moderate differentiated	67	77.9
	Poorly differentiated	13	15.1
T Invasion	T1	5	5.8
	T2	21	24.4
	T3	47	54.7
	T4	13	15.1
LN	N0	51	59.3
	N1	21	24.4
	N2	14	16.3
Metastasis	M0	81	94.2
	M1	5	5.8
Stage	I	16	18.6
	II	31	36
	III	34	39.5
	IV	5	5.8
Histological type	Adenocarcinoma	75	87.2
	Mucinous adenocarcinoma	11	12.8
Neural invasion	Positive	4	4.7
	Negative	82	95.3
Vascular invasion	Positive	64	74.4
	Negative	22	25.6
Intraepithelial neoplasia nearby	Positive	67	77.9
	Negative	19	22.1

Regarding the tumor invasion, T3 (54.7%) (47) was the most common invasion depth. Lymph node involvement shows absence of invasion in (59.3%) of 51 cases. Stage II 31 (36%) and III 34 (39.5%) had substantial representation, whereas in stages I 16 (18.6%) and IV only five (5.8%) were less prevalent. Adenocarcinoma (87.2%) (75) cases were the dominant histological type. Neural invasion was detected in a minority (four) (4.7%), while vascular invasion (64) (74.4%) and intraepithelial neoplasia (67) (77.9%) were more common.

Association of different markers with clinicopathological features

The association between PTEN and K-ras downstream protein expression with clinicopathological features was investigated.

Tumor location revealed interesting patterns. Patients with tumors located in the right colon exhibited a significant association between tumor location and PTEN, and ERK expressions respectively (P= 0.031 and P=0.009), suggesting a potential role of PTEN and ERK in specific colon segments. However, no other consistent significant associations were observed in relation to AKT, or P65 expression.

The type of tumor growth yielded no consistent significant associations with PTEN, AKT, ERK, or P65 expression. Interestingly, the size of the tumor was significantly associated with P65 expression (P=0.042), potentially suggesting a role for P65 in tumor growth. There were significant associations between T invasion and P65 expression (P=0.026).

Also, the perineural invasion displayed a significant association with PTEN expression (P=0.045), suggesting a potential role of PTEN in neural invasion inhibition. Notably, intraepithelial neoplasia nearby demonstrated a significant association with AKT expression (P=0.017), indicating potential connections between AKT activation and the presence of intraepithelial neoplasia.

However, the other parameters exhibited no consistent significant associations with PTEN, AKT, ERK, or P65 expression (Table 3).

**Table 3:** The table presents an in-depth analysis of the associations between clinicopathological features and the expression levels of PTEN, AKT, ERK, and P65 markers. *: p<0.05 is considered significant no. = number of cases, % = 100 percentage

Clinicopathological parameters		PETN no. (%)		AKT no. (%)		ERK no. (%)		P65 no. (%)		Total 86 cases
		Positive	Negative	Positive	Negative	Positive	Negative	Positive	Negative	
Tumor location	Right colon	16 (37.2)	27 (62.8)	29 (67.4)	14 (32.6)	13 (30.2)	30 (69.8)	22 (51.2)	21 (48.8)	43
	Left colon	26 (60.5)	17 (39.5)	27 (62.8)	16 (37.2)	25 (58.1)	18 (41.9)	30 (69.8)	13 (30.2)	43
p-value		0.031^*^		0.651		0.009^*^		0.078		
Type of growth	Polypoid	16 (44.4)	20 (55.6)	23 (65.7)	13 (24.3)	14 (38.9)	22 (61.1)	19 (52.8)	17 (47.2)	36
	Ulcerated	19 (54.3)	16 (45.7)	21 (60.0)	14 (40.0)	18 (51.4)	17 (48.6)	26 (74.3)	9 (25.7)	35
	Circumferential	3 (37.5)	5 (62.5)	6 (75.0)	2 (25.0)	4 (50.0)	4 (50.0)	3 (37.5)	5 (62.5)	8
	Fungating	4 (57.1)	3 (42.9)	6 (85.7)	1 (14.3)	2 (28.6)	5 (71.4)	4 (57.1)	3 (42.9)	7
p-value		0.729		0.556		0.582		0.14		
Size of tumor	≤5 cm	22 (46.8)	25 (53.2)	31 (66.0)	16 (34.0)	17 (36.2)	30 (63.8)	33 (70.2)	14 (29.8)	47
	>5 cm	20 (51.3)	19 (48.7)	25 (64.1)	14 (35.9)	21 (53.8)	18 (46.2)	19 (48.7)	20 (51.3)	39
p-value		0.679		0.857		0.1		0.042^*^		
Grade	Well-differentiated	1 (16.7)	5 (83.3)	2 (33.3)	4 (66.7)	3 (50.0)	3 (50.0)	4 (66.7)	2 (33.3)	6
	Moderate differentiated	37 (55.2)	30 (44.8)	43 (64.2)	24 (35.8)	31 (46.3)	36 (53.7)	43 (64.2)	24 (35.8)	67
	Undifferentiated	4 (30.8)	9 (69.2)	11 (84.6)	2 (15.4)	4 (30.8)	9 (69.2)	5 (38.5)	8 (61.5)	13
p-value		0.071		0.088		0.563		0.211		
Stage	I	6 (35.3)	11 (64.7)	7 (41.2)	10 (58.8)	6 (35.3)	11 (64.7)	7 (41.2)	10 (58.8)	17
	II	19 (61.9)	12 (38.1)	21 (67.7)	10 (32.3)	17 (54.8)	14 (45.2)	24 (77.4)	7 (22.6)	31
	III	15 (45.5)	18 (54.5)	23 (69.7)	10 (30.3)	13 (39.4)	20 (60.6)	17 (51.5)	16 (48.5)	33
	IV	2 (40.0)	3 (60.0)	5 (100)	0.0	2 (40.0)	3 (60.0)	4 (80.0)	1 (20.0)	5
p-value		0.323		0.061		0.51		0.041^*^		
T Invasion	T1	1 (20.0)	4 (80.0)	2 (40.0)	3 (60.0)	4 (80.0)	1 (20.0)	3 (60.0)	2 (40.0)	5
	T2	11 (52.4)	10 (47.6)	13 (61.9)	8 (38.1)	8 (38.1)	13 (61.9)	7 (33.)	14 (66.7)	21
	T3	24 (51.1)	23(48.9)	31 (65.9)	16 (34.1)	22 (46.8)	25 (53.2)	34 (72.3)	13 (27.7)	47
	T4	6 (46.2)	7 (53.8)	10 (76.9)	3 (23.1)	4 (30.8)	9 (69.2)	8 (61.5)	5 (38.5)	13
p-value		0.59		0.51		0.62		0.026^*^		
LN	N0	27 (52.9)	24 (47.1)	31 (60.8)	20 (39.2)	25 (49.0)	26 (51.0)	32 (62.7)	19 (37.3)	51
	N1	9 (42.9)	12 (57.1)	15 (71.4)	6 (28.6)	10 (47.6)	11 (52.4)	11 (52.4)	10 (47.6)	21
	N2	6 (42.9)	8 (57.1)	10 (71.4)	4 (28.6)	3 (21.4)	11 (78.6)	9 (64.3)	5 (35.7)	14
p-value		0.656		0.596		0.172		0.68		
Metastasis	M0	40 (49.4)	41 (50.6)	51 (62.9)	30 (37.1)	36 (44.4)	45 (55.6)	48 (59.3)	33 (40.7)	81
	M1	2 (40.0)	3 (60.0)	5 (100)	0.0	2 (40.0)	3 (60.0)	4 (80.0)	1 (20.0)	5
p-value		0.684		0.092		0.846		0.357		
Histological type	Adenocarcinoma	38 (50.7)	37 (48.3)	49 (65.3)	26 (34.7)	32 (42.7)	43 (57.3)	45 (60.0)	30 (40.0)	75
	Mucinous adenocarcinoma	4 (36.4)	7 (63.6)	7 (63.6)	4 (36.4)	6 (54.5)	5 (45.5)	7 (63.6)	4 (36.4)	11
p-value		0.375		0.912		0.459		0.818		
Neural invasion	Positive	0.0	4 (100)	3 (75.0)	1 (25.0)	2 (50.0)	2 (50.0)	4 (100)	0.0	4
	Negative	42 (51.2)	40 (48.8)	53 (64.6)	29 (35.4)	36 (43.9)	46 (56.1)	48 (58.5)	34 (41.5)	82
p-value		0.045^*^		0.67		0.81		0.098		
Vascular invasion	Positive	32 (50.0)	32 (50.0)	42 (65.6)	22 (34.4)	25 (39.1)	39 (60.9)	40 (62.5)	24 (37.5)	64
	Negative	10 (45.5)	12 (54.5)	14 (63.6)	8 (36.4)	13 (59.1)	9 (40.9)	12 (54.5)	10 (45.5)	22
p-value		0.713		0.866		0.103		0.51		
Intra-epithelial neoplasia nearby	Positive	33 (49.3)	34 (50.7)	48 (71.6)	19 (28.4)	27 (40.3)	40 (59.7)	39 (58.2)	28 (41.8)	67
	Negative	9 (47.4)	10 (52.6)	8 (42.1)	11 (57.9)	11 (57.9)	8 (42.1)	13 (68.4)	6 (31.6)	19
p-value		0.885		0.017^*^		0.173		0.422		

Relationship between PTEN expression and markers

Regarding ERK marker expression, individuals with high PTEN expression demonstrated a higher prevalence of high ERK expression 24 (57.14%) compared to those with low PTEN expression 14 (31.82%). This difference was statistically significant, with a P-value of 0.018. This suggests that there might be a relationship between PTEN and ERK signaling, indicating a possible regulatory role of PTEN in the ERK pathway.

For the AKT marker expression, individuals with high PTEN expression exhibited a higher frequency of high AKT expression 32 (76.19%) compared to those with low PTEN expression 24 (54.55%). This difference reached statistical significance, with a P-value of 0.035. This finding suggests that PTEN plays a role in influencing the activation of the AKT pathway.

**Figure 1:**
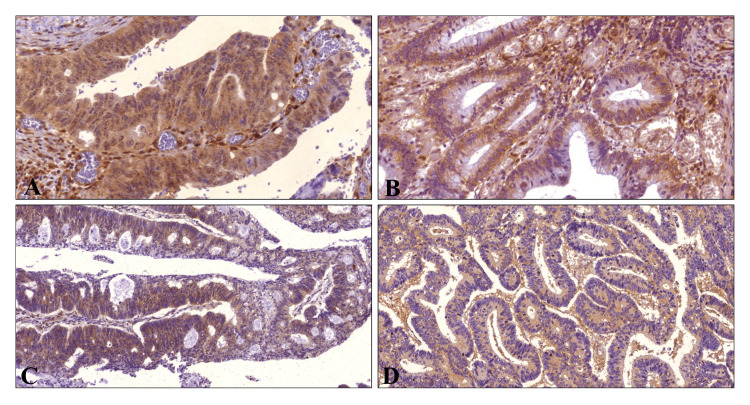
Expression and localization of the markers in colonic carcinoma. PTEN (A), AKT (B), and ERK (C) show intense nuclear expression and expression in the cytoplasm, respectively. P65 (D) shows cytoplasmic expression (x100).

In the case of the P65 marker, both high and low PTEN expression groups displayed similar patterns of P65 marker expression. The differences observed were not statistically significant (P=0.479), suggesting that PTEN expression might not have a strong influence on P65 activation. These results reflect that PTEN expression could be blocked and associated with the expression of ERK and AKT markers. High PTEN expression appears to correlate with increased ERK and AKT marker expression, suggesting a potential regulatory role of PTEN in these pathways. However, PTEN expression does not seem to significantly impact P65 marker expression. These findings contribute to our understanding of the intricate interactions between PTEN and various signaling pathways, potentially guiding future research and therapeutic strategies aimed at modulating these pathways in the context of cancer and other diseases (Figure 1).

The data presented in Table 4 outlines the relationship between PTEN expression and the expression levels of the signaling markers ERK, AKT, and P65.

**Table 4:** Relationship between PTEN expression and ERK, AKT, and P65 *: P<0.05 is considered significant no. = number of cases, % = 100 percentage

Markers expression		PTEN expression		Total 86 Cases	P-Value
		Positive no. (%)	Negative no. (%)		
ERK	Positive	24 (57.14)	18 (42.86)	42	0.018^*^
	Negative	14 (31.82)	30 (68.18)	44	
AKT	Positive	32 (76.19)	10 (23.81)	42	0.035*
	Negative	24 (54.55)	20 (45.45)	44	
P65	Positive	27 (64.29)	15 (35.71)	42	0.479
	Negative	25 (56.82)	19 (43.18)	44	

KRAS mutation features in CRC

**Figure 2:**
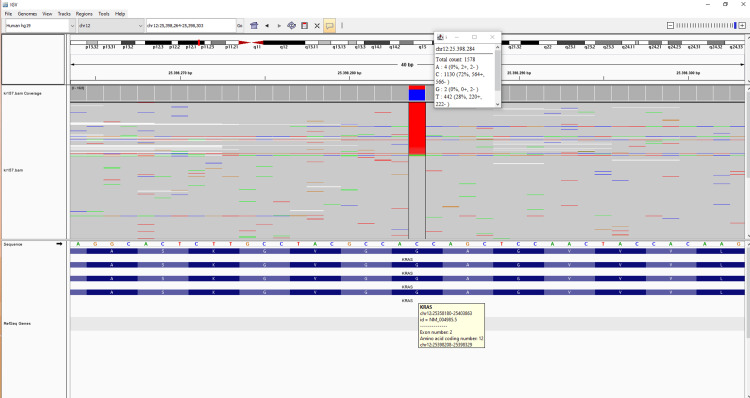
Integrative Genomics Viewer (IGV) images of next-generation sequencing (NGS) data of KRAS c.35G>A (p.Gly12Asp) variant detected in the DNA of embedded tissue sample (J H2850).

Table 5 shows a list of mutations in the KRAS gene, providing details of mutation type, amino acid changes, allele frequencies, codon and exon numbers. Out of 86 cases of CRC, 32 cases (37.2%) exhibited mutation of the KRAS gene. All mutations were of missense type. The allele frequencies are widely variable, indicating different prevalence levels for each mutation within the sample. For instance, mutations like c.34G>T (p.Gly12Cys) and c.35G>C (p.Gly12Ala) have high allele frequencies of 54% and 47%, respectively, while others like c.182A>G (p.Gln61Arg) and c.287A>G (p.Tyr96Cys) have lower frequencies of 3% and 5%; respectively. Some mutations, such as c.35G>A (p.Gly12Asp), c.35G>T (p.Gly12Val), c.38G>A (p.Gly13Asp), and c.34G>T (p.Gly12Cys), appear more frequent. Certain codons (e.g., 12 and 13) appear to have mutation hotspots, about 53.1% and 12.5% of mutant cases respectively. The exon 2 was the most common mutant exon around 75% followed by exon 3 about 18.75% (Figure 2).

**Table 5:** Frequency of KRAS mutation in CRC no. is the number of cases, % is allele frequency from 100 percentage, CRC: colorectal cancer

No.	KRAS mutant	Mutation type	Amino acid change	Exon Number	Allele frequency	ClinVar
1	c.35G>T	missense	(p.Gly12Val)	2	44%	12583
2	c.35G>T	missense	(p.Gly12Val)	2	16%	12583
3	c.38G>A	missense	(p.Gly13Asp)	2	29%	12580
4	c.38G>A	missense	(p.Gly13Asp)	2	4%	12580
5	c.95A>T	missense	(p.Tyr32Phe)	2	13%	novel variant
6	c.182A>G	missense	(p.Gln61Arg)	3	3%	45115
7	c.34G>T	missense	(p.Gly12Cys)	2	29%	12578
8	c.38G>A	missense	(p.Gly13Asp)	2	32%	12580
9	c.34G>T	missense	(p.Gly12Cys)	2	54%	12578
10	c.38G>A	missense	(p.Gly13Asp)	2	12%	12580
11	c.187G>A	missense	(p.Glu63Lys)	3	5%	2498256
12	c.35G>C	missense	(p.Gly12Ala)	2	47%	45122
13	c.440A>G	missense	(p.Lys147Arg)	4	14%	431103
14	c.169G>A	missense	(p.Asp57Asn)	3	3%	COSM1166779
15	c.287A>G	missense	(p.Tyr96Cys)	3	5%	novel variant
16	c.13A>G	missense	(p.Lys5Glu)	2	15%	12596
17	c.35G>C	missense	(p.Gly12Ala)	2	33%	45122
18	c.35G>A	missense	(p.Gly12Asp)	2	24%	12582
19	c.34G>A	missense	(p.Gly12Ser)	2	8%	12584
20	c.34G>A	missense	(p.Gly12Ser)	2	5%	12584
21	c.440A>G	missense	(p.Lys147Arg)	4	7%	431103
22	c.35G>A	missense	(p.Gly12Asp)	2	24%	12582
23	c.35G>A	missense	(p.Gly12Asp)	2	52%	12582
24	c.35G>A	missense	(p.Gly12Asp)	2	28%	12582
25	c.216G>A	missense	(p.Met72Ile)	3	6%	132970
26	c.35G>A	missense	(p.Gly12Asp)	2	42%	12582
27	c.35G>T	missense	(p.Gly12Val)	2	3%	12583
28	c.13A>G	missense	(p.Lys5Glu)	2	61%	12596
29	c.216G>A	missense	(p.Met72Ile)	3	12%	132970
30	c.35G>T	missense	(p.Gly12Val)	2	8%	12583
31	c.35G>A	missense	(p.Gly12Asp)	2	38%	12582
32	c.35G>T	missense	(p.Gly12Val)	2	26%	12583

Association of KRAS mutation status with clinicopathological characteristics

The relationship between KRAS mutation status and various clinicopathological features is shown in Table 6. The KRAS molecular study revealed (37.2%) 32 cases positive and (62.8) 54 cases were negative. Interestingly, a significant relationship was observed between KRAS mutation status and the type of tumor growth. Polypoid and circumferential growth patterns exhibited a higher incidence of KRAS mutations, whereas ulcerated and fungating growth patterns showed exclusively fewer mutant KRAS cases (p-value = 0.035). Tumor grade displayed an association with KRAS mutations. Well-differentiated tumors showed a lower frequency of KRAS mutations compared to moderate and poorly differentiated tumors (p-value = 0.043).

**Table 6:** KRAS mutation with clinicopathological features *: p<0.05 is considered significant no. = number of cases, % = 100 percentage

Variables		KRAS Mutation		p-value	Total cases
		Mutant no. (%)	wild no. (%)		
Tumor location	Right colon	17 (39.5)	26 (60.5)	0.655	43
	Left colon	15 (34.9)	28 (65.1)		43
Type of growth	Polypoid	18 (50.0)	18 (50.0)	0.035*	36
	Ulcerated	10 (28.6)	25 (71.4)		35
	Circumferential	4 (50.0)	4 (50.0)		8
	Fungating	0 (0.0)	7 (100)		7
Size of tumor	≤5	17 (36.2)	30 (63.8)	0.827	47
	>5	15 (38.5)	24 (61.5)		39
Grade	Well-differentiated	2 (33.3)	4 (66.7)	0.043*	6
	Moderate differentiated	29 (43.3)	38 (56.7)		67
	Poorly differentiated	1 (7.7)	12 (92.3)		13
Stage	I	8 (47.1)	9 (52.9)	0.213	17
	II	13 (41.9)	18 (58.1)		31
	III	8 (24.2)	25 (75.8)		33
	IV	3 (60.0)	2 (40.0)		5
T invasion	T1	3 (60.0)	2 (40.0)	0.52	5
	T2	8 (38.1)	13 (61.9)		21
	T3	18 (38.3)	29 (61.7)		47
	T4	3 (23.1)	10 (76.9)		13
LN	N0	22 (43.1)	29 (56.9)	0.141	51
	N1	4 (19.0)	17 (81.0)		21
	N2	6 (42.9)	8 (57.1)		14
Metastasis	M0	29 (35.8)	52 (64.2)	0.277	81
	M1	3 (60.0)	2 (40.0)		5
Histological type	Adenocarcinoma	27 (36.0)	48 (64.0)	0.545	75
	Mucinous adenocarcinoma	5 (45.50	6 (54.5)		11
Perineural invasion	Positive	3 (75.0)	1 (25.0)	0.109	4
	Negative	29 (35.4)	53 (64.6)		82
Vascular invasion	Positive	24 (37.5)	40 (62.5)	0.924	64
	Negative	8 (36.4)	14 (63.60		22
Intraepithelial neoplasia nearby	Positive	24 (35.8)	43 (64.2)	0.617	67
	Negative	8 (42.1)	11 (57.9)		19

However, no significant association was observed between KRAS mutation and other clinicopathological features including tumor location, tumor size, stage, lymph node involvement, metastasis, adenocarcinoma and mucinous adenocarcinoma, neural invasion, vascular invasion and intraepithelial neoplasia nearby.

Relationship between KRAS mutation with AKT, ERK, and P65 markers

The data presented in Table 7 illustrates the correlation between KRAS mutation and the expression levels of the signaling markers ERK, AKT, and P65. The analysis aimed to uncover potential associations between these markers and the occurrence of KRAS mutations in the studied population.

**Table 7:** KRAS mutation with AKT, ERK, and P65 markers p<0.05 is considered significant no. = number of cases, % = 100 percentage

Markers expression		KRAS mutation		Total 86 cases	P-Value
		Positive no. (%)	Negative no. (%)		
ERK	Positive	17 (53.12)	15 (46.88)	32	0.199
	Negative	21 (38.9)	33 (61.1)	54	
AKT	Positive	22 (68.75)	10 (31.25)	32	0.586
	Negative	34 (62.96)	20 (37.04)	54	
P65	Positive	24 (75.0)	8 (25.0)	32	0.034*
	Negative	28 (51.85)	26 (48.15)	54	

In terms of ERK marker expression, individuals with high expression demonstrated a higher prevalence of positive KRAS mutations 17 (53.12%) compared to those with low expression 21 (38.9%). However, the observed difference was not statistically significant (P=0.199), suggesting that ERK expression may not be a decisive factor in determining KRAS mutation status.

Similarly, AKT marker expression revealed that individuals with high expression had a higher frequency of positive KRAS mutations 22 (68.75%) compared to those with low expression 34 (62.96%). Nonetheless, the observed difference did not reach statistical significance (P=0.586), indicating that AKT expression might not be a strong predictor of KRAS mutation presence.

In contrast, the P65 marker displayed a notable pattern. Individuals with high expression of the P65 marker exhibited a significantly higher likelihood of having positive KRAS mutations 24 (75.0%) in comparison to those with low P65 expression 28 (51.85%), with a statistically significant P-value of 0.034. This finding suggests a potential association between P65 expression and the presence of KRAS mutations, indicating that P65 may play a role in KRAS-driven oncogenic processes.

## Discussion

The present data provides a detailed snapshot of the clinicopathological parameters of CRC. These information are instrumental in understanding the characteristics of the patient cohort and they are vital for both clinical decision-making and further research in the field of CRC.

The patient distribution by sex is relatively balanced, with slightly more males 46 (53.5%) than females 40 (46.5%), reflecting CRC's impact on both genders. A significant majority of patients 57 (66.3%) were aged 50 or below, emphasizing the importance of early screening. Tumors were found in various parts, with the highest frequencies in the cecum 29 (33.7%), sigmoid colon 21 (24.4%), and rectum 19 (22.1%). Tumor growth and size data show nearly half exhibiting polypoid 36(41.9%) or ulcerated 35 (40.7%) patterns, influencing tumor aggressiveness and surgical approaches.

Adenocarcinomas 75 (87.2%) are the predominant histological type, with mucinous adenocarcinomas 11 (12.8%) present. Tumor grade indicates differentiation, with the majority moderately differentiated 67 (77.9%) and a smaller proportion poorly and well-differentiated 13 and six (15.1% and 7%) respectively. Tumor invasion data shows most cases at T3 47 (54.7%), indicating infiltration through the colon wall. Lymph node involvement showed a high rate of absence of infiltration in 51 (N0, 59.3%) and absence of distant metastasis in 81 (M0, 94.2%) of cases suggesting early-stage diagnoses.

The distribution across cancer stages reveals many cases at stage II 31 (36%) and stage III 34 (39.5%), indicating locally advanced disease at diagnosis. These findings highlight a comprehensive reference for CRC parameters, aiding clinicians and guiding further research into the disease's complexities.

The association between clinicopathological features and the expression levels of PTEN, AKT, ERK, and P65 markers offer valuable insights into potential associations that may help unravel the underlying mechanisms involved in CRC development and progression.

Tumor location emerged as an intriguing factor in relation to marker expression. Significant associations were found between PTEN, ERK expressions, and tumor location in the right colon. This could indicate a potential role of PTEN and ERK signaling in specific colon segments, potentially influencing tumor behavior regarding the role of both markers in tumor initiation. Furthermore, we found no significant associations between AKT and P65 expressions and tumor location. Certain investigators observed that there were no statistically significant variances in PTEN expression concerning the tumor's location in the colon [16]. Nevertheless, others noted that PTEN expression was lower in distal tumors in comparison to proximal tumors [17].

Type of tumor growth, including ulcerated, polypoid, fungating, and circumferential growth patterns, displayed no consistent correlations with marker expression. This suggests that the activation of PTEN, AKT, ERK, and P65 signaling pathways might not be heavily influenced by the macroscopic characteristics of the tumor. PTEN expression exhibited similarity in the primary tumor mass [16].

Tumor size exhibited a significant association with P65 expression. This finding may hint at the potential involvement of P65 signaling in the growth and development of larger tumors. It has been proposed that the cytoplasmic expression of NF-κB/p65 may have a significant impact on the progression of CRC, potentially through molecular alterations in its downstream pathway. Furthermore, prior investigations have suggested a positive connection between the expression of NF-κB/p65 and histopathological differentiation, tumor depth, lymph node involvement, and the extent of metastasis. Notably, there is no established link between NF-κB/p65 expression and tumor localization in these instances. It is important to highlight that the complete scope of NF-κB/p65's correlation with clinicopathological parameters remains to be thoroughly elucidated [13].

The grade and stage of cancer are essential determinants of prognosis. Our analysis demonstrated trends, albeit non-significant, between marker expression and tumor grade and stage. These trends suggest that PTEN, AKT, ERK, and P65 signaling pathways could play roles in tumor differentiation and progression, warranting further investigations.

Invasion depth (T stage) and lymph node involvement (N stage) are key indicators of cancer aggressiveness. The observed trends and significant associations between marker expression and T stage or N stage underscore the potential significance of PTEN, AKT, ERK, and P65 pathways in these aspects of tumor behavior. PTEN expression showed similarity in the primary lymph node metastasis [18].

Histological type and other factors like neural and vascular invasion demonstrated intriguing relationships with marker expression, indicating the potential roles of these signaling pathways in specific tumor characteristics and interactions with the tumor microenvironment.

Notably, the presence of intraepithelial neoplasia nearby displayed a significant association with AKT expression. This finding suggests that the AKT signaling pathway might be implicated in the progression from intraepithelial neoplasia to invasive cancer.

The associations between clinicopathological features and the expression of PTEN, AKT, ERK, and P65 markers provide a basis for further investigations into the precise roles of these signaling pathways in various stages of CRC development, aiding in the understanding of potential therapeutic targets and prognostic markers.

The analysis revealed distinct patterns of associations, exhibiting high expression levels of both ERK and AKT markers were significantly more likely to display elevated PTEN expression when compared to those with low ERK and AKT expression, with percentages of 57.14% (24) and 31.82% (14), and 76.19% (32) and 54.55% (24) respectively. The statistically significant differences (P=0.018 and P=0.035) suggest a potential regulatory role of ERK and AKT in influencing PTEN expression levels. These findings contribute to our understanding of the intricate interplay between these signaling components and the intricate regulatory mechanisms that govern them. PTEN's capacity to counteract neo-angiogenesis seems to be associated with its lipid phosphatase activity and its ability to suppress signaling through the PI3K/AKT/mTOR pathway [19]. The loss of PTEN expression could potentially serve as an indicator of the response to treatments targeting the PI3K/Akt pathway [20]. Some researchers have noted less uniform associations between heightened p-AKT expression and the activation of downstream elements within the pathway when contrasted with PTEN and AKT mutations [21]. In our investigation, we identified a robust and statistically significant correlation between the expressions of AKT and PTEN and the activation of downstream components, attaining a noteworthy p-value of 0.035.

In contrast, the study did not reveal a significant difference in the prevalence of high P65 marker expression between patients with high and low PTEN expression, with percentages of 64.29% (27) and 56.82% (25) respectively (P=0.479). This suggests that the influence of PTEN on P65 expression might not be as pronounced, emphasizing the need for further exploration of their potential interactions.

Overall, these results underscore the intricate relationships between PTEN and signaling markers, hinting at specific roles in regulating ERK and AKT pathways. This complexity offers promise for advancing our understanding of cancer mechanisms and lays the groundwork for targeted therapeutic strategies based on individual molecular profiles.

This study reveals the intricate relationship between KRAS mutation and signaling markers (ERK, AKT, P65) in cancer. Elevated P65 expression is significantly associated with a positive KRAS mutation. Loss of PTEN functions is crucial in many cancers, particularly RAS-driven ones [19]. The NF-κB (p65) signaling pathway is vital for promoting cell survival, particularly in RAS-driven cancers [22]. Where increased RAL, ERK, and AKT activation occurs even without RAS mutation [23]. The focus here is on the association between loss of PTEN expression and the P65 pathway, highlighting P65's potential role in driving oncogenic processes in the presence of KRAS. Further investigation is needed to understand its mechanisms.

Furthermore, the study found a comparable prevalence of positive KRAS mutation in patients with high and low ERK and AKT marker expression. Although not statistically significant, subtle differences suggest nuanced interactions requiring deeper exploration. The influence of PTEN status on RAS/RAF/MAPK sensitivity and its potential as an indicator of resistance to targeted therapies, such as those targeting anti-VEGF (vascular endothelial growth factor) and drugs aimed at the PI3K/AKT pathway, is under scrutiny [20].

The correlation between KRAS mutation status and various clinicopathological features contributes to a deeper understanding of the potential role of KRAS mutations in tumor development and progression, shedding light on their relationship with different aspects of the disease. The tumor location analysis did not reveal a significant association between KRAS mutations and the colon's right or left side (p-value = 0.655). This result suggests that KRAS mutations are not preferentially associated with a specific colon segment.

The most striking finding in the study is the significant correlation between KRAS mutations and the type of tumor growth (p-value = 0.035). Specifically, the polypoid growth pattern exhibited a higher incidence of KRAS mutations, and cases with fungating growth exclusively exhibited mutant KRAS. This association could suggest that KRAS mutations might influence certain tumor patterns of growth. The analysis of tumor size revealed no significant correlation with KRAS mutations (p-value = 0.827), indicating that tumor size may not be strongly influenced by KRAS mutational status.

The distribution of KRAS mutations did not significantly differ between adenocarcinoma and mucinous adenocarcinoma (p-value = 0.545). This implies that KRAS mutations might not be the primary determinant of histological subtype. Tumor grade displayed a statistically significant relationship with KRAS mutations (p-value = 0.043), with well-differentiated tumors exhibiting fewer mutations compared to moderate and undifferentiated tumors. Regarding the invasion, there is an absence of significant associations between KRAS mutations and tumor stage (p-value = 0.213) as well as T invasion depth (p-value = 0.52). There was no significant correlation between KRAS mutations and lymph node involvement (p-value = 0.141). Similarly, the analysis revealed no significant association between KRAS mutations and the presence of metastasis (p-value = 0.277). This suggests that the presence of KRAS mutations might play a role in tumor differentiation and may not be a strong determinant of tumor invasion and metastasis, indicating that other factors might contribute more substantially to metastatic potential.

This research provides nuanced insights into the multifaceted nature of KRAS-associated oncogenesis, highlighting the importance of considering various signaling pathways in understanding cancer development. These findings contribute to our understanding of KRAS mutations' molecular landscape and suggest potential avenues for targeted therapeutic interventions. As we explore the complex network of signaling interactions in cancer progression, these findings encourage tailored treatment strategies based on individual tumor molecular characteristics, potentially advancing precision medicine and improving patient outcomes in cancer therapy.

Limitations

While the study offers crucial insights into the relationships among PTEN expression, KRAS mutation, and downstream pathways in CRC, it has some limitations: The findings rely on a specific sample set, potentially not fully capturing CRC heterogeneity, other gene mutations, and interactions with other key factors. It is a cross-sectional design and does not cover the long-term outcome prediction, especially the prognosis and survival rate in correlation with the findings of this study. Emphasizing the need for further comprehensive studies to validate and expand on these initial observations.

## Conclusions

The study establishes a significant correlation between PTEN expression and the inhibition of AKT and ERK, particularly in the absence of the KRAS gene mutation. However, a divergent relationship emerges when considering PTEN expression with P65 and a KRAS mutation. In instances where PTEN is not expressed, a positive correlation is observed with P65, and the presence of a KRAS mutation is notable. The intricate delineation reveals complex dynamics in CRC, emphasizing the interplay between PTEN expression, KRAS mutation, and downstream signaling pathways. These findings highlight the intricate complexity of molecular interactions in CRC progression and suggest potential targets for therapeutic interventions, paving the way for future research initiatives and optimized treatment strategies.
